# A PCR-based forward genetics screening, using expression domain-specific markers, identifies mutants in endosperm transfer cell development

**DOI:** 10.3389/fpls.2014.00158

**Published:** 2014-04-28

**Authors:** Luis M. Muñiz, Elisa Gómez, Virginie Guyon, Maribel López, Bouchaib Khbaya, Olivier Sellam, Pascual Peréz, Gregorio Hueros

**Affiliations:** ^1^Departamento Biomedicina and Biotecnología (Genética), Universidad de AlcaláAlcalá de Henares, Spain; ^2^GM Trait Discovery, Biogemma, Centre de Recherche de ChappesChappes, France

**Keywords:** transfer cells, development, aleurone, mutant, gene expression

## Abstract

Mutant collections are an invaluable source of material on which forward genetic approaches allow the identification of genes affecting a wide variety of biological processes. However, some particular developmental stages and morphological structures may resist analysis due to their physical inaccessibility or to deleterious effects associated to their modification. Furthermore, lethal mutations acting early in development may escape detection. We have approached the characterization of 101 maize seed mutants, selected from a collection of 27,500 visually screened Mu-insertion lines, using a molecular marker approach based on a set of genes previously ascribed to different tissue compartments within the early developing kernel. A streamlined combination of qRT-PCR assays has allowed us to preliminary pinpoint the affected compartment, establish developmental comparisons to WT siblings and select mutant lines with alterations in the different compartments. Furthermore, clusters of markers co-affected by the underlying mutation were identified. We have analyzed more extensively a set of lines presenting significant variation in transfer cell-associated expression markers, and have performed morphological observations, and immunolocalization experiments to confirm the results, validating this approach as an efficient mutant description tool.

## Introduction

The angiosperm seed is a complex structure evolutionarily designed to attain protection of the forming embryo, resist desiccation, facilitate its dispersal and nurture the new plant before it obtains sufficient photosynthetic ability for self-support. The synchronized formation of tissular domains and activation of their different functions presents a very interesting scenario for developmental studies (Berger, 2003), as plant seeds are easy to obtain in great numbers in a controlled manner, dissection and structure observation is easy, and similar developmental patterns are implemented with little variation by very different plant species. A number of works have described the architecture and development of the seed (Wobus and Weber, 1999; Boisnard-Lorig et al., 2001; Thompson et al., 2001). Among these, the maize seed has received particular attention due to its economic value and widespread cultivation (Timmermans et al., 2004). It is the result of a double fertilization event which produces two isolated compartments differing in ploidy level but otherwise genetically identical, the embryo and the endosperm. The endosperm develops from the fusion of one sperm nucleus with the central cells of the embryo sac, producing a triploid syncytial tissue which follows a nuclear type of development (Kiesselbach, 1949). Few days after pollination, active cell division yields to a differentiation process that results in four specialized tissues within the endosperm (Olsen, 2001; Consonni et al., 2005; Scanlon and Takacs, 2008): the starchy endosperm, which accumulates nutrients for seedling nutrition after germination; the ESR, presumably involved in nutrient transfer from the mother plant to the embryo and its protection (Balandín et al., 2005); the basal endosperm transfer layer (BETL), located right under the starchy endosperm and opposite to the placento-chalaza, where it facilitates solute uptake and secretes defensive peptides onto the seed-mother plant interface (Serna et al., 2001; Olsen, 2004); and the aleurone, a peripheral layer of nearly cubical cells which participates in substrate mobilization upon germination of the embryo (Bommert and Werr, 2001) and covers the entire surface of the endosperm except for the BETL.

Forward genetics is a time-proven, reliable strategy to identify genes involved in the regulation of biological processes. Providing researchers with a collection of individuals on which to perform phenotypic screenings and amenable to an easy search for the causative gene has been the goal of an increasing number of mutant collections in several biological systems (*Arabidopsis*; Alonso et al., 2003, maize; Walbot, 2000, rice; An et al., 2005, *Drosophila*; Bellen et al., 2004, *Caenorhabditis*; Barstead and Moerman, 2006, mouse; Adams et al., 2004…). The study of early developmental processes or tissue differentiation through mutant screening poses however some problems, like the reduced size and accessibility of some tissues and the loss of individuals bearing defects deleterious for organ/tissue organization in early stages (Warren and Fishman, 1998). Technologies able to detect and quantify markers for development and function in minute samples and/or in a fast, simple manner may open the possibility to scrutinize tissue organization and functionality in a reliable way (Borisjuk et al., 1998), thus facilitating the identification of interesting mutants. Genes expressed in a tissue-specific manner are thus excellent candidates as tools to detect mutations affecting cell fate and organ determination.

Genetic determinants and markers for the endosperm differentiated tissues have been described along the years (Opsahl-Ferstad et al., 1997; Bonello et al., 2000; Becraft, 2001; Gómez et al., 2002). Many of these genes have been shown to be intimately associated to the presence/function of a specific cell type and may provide an unequivocal beacon of its presence or state of development. Additionally, genes with known expression kinetics along tissue differentiation may allow pinpointing precisely the moment when a given mutation is relevant for tissue identity or function. To test the feasibility of a molecular-based analysis of the maize seed as mutant-screening tool, we have analyzed 101 maize mutant lines showing alterations in the kernel development via a streamlined combination of qRT-PCR tests. We have described the molecular effect of the mutations in each line on kernel formation, pinpointed the affected cell types according to the expression patterns, and validated the results by performing histological descriptions and immunolocalization of sibling kernels, confirming this approach as an efficient mutant screening tool.

## Materials and methods

### Plant material

One hundred and one maize lines showing monogenic segregation for mutations affecting kernel development were selected from a collection of 27,500 Mutator-mutagenized maize lines (Martin et al., 2006). Wild type (WT) and mutant sibling kernels were dissected out from segregating ears once they could be un-ambiguously distinguished (in most cases between 13 and 15 DAP). Kernels were immediately frozen in liquid N_2_ and stored at −80°C until they were used for RNA extraction.

### RNA extraction

RNA was extracted from 2 WT and 2 mutant kernels for each line using the FastRNA® Pro System (Bio101) with minor modifications to the manufacturer's protocol. Briefly, frozen seeds were crushed in liquid nitrogen and immediately mixed with lysis buffer. After completing the extraction and prior to quantitation, the RNA solutions were frozen at −80°C and centrifuged to precipitate starch.

### qRT-PCR design

At least one of the primers selected for each gene spanned an intron site in the genomic DNA, to avoid amplification of contaminating DNA in the RNA preparations. Primer couples were designed to amplify short cDNA sequences to maximize the efficiency of the PCR reactions.

The qRT-PCR reactions were performed in an ABI-PRISM-7000 instrument using the Power SYBR Green One-Step RT-PCR Master Mix, both from Applied Biosystems, and 20 ng of total RNA as template. Reactions were checked for specificity by visual inspection of melting curves and relative expression values were calculated using the ΔΔCt method, normalizing to the *ZmFKBP-66* gene Ct in the same sample and comparing the WT/mutant pairs.

### Immunolocalization and histology

Seeds kept at −80°C were hand dissected whilst frozen and immediately fixed in 0.1 M phosphate buffer pH 7.2 plus 4% paraformaldehyde, 0.1% glutaraldehyde. Samples were then dehydrated in an ethanol series and embedded in Paraplast (Sigma). Sections 8 μm thick were attached to sylanized glass slides. Slides were deparaffinised with xylene (Dimethyl-benzene) and then rehydrated in an ethanol series. Endogenous peroxidase activity was deactivated by incubation in 0.3% hydrogen peroxide for 20 min. Sections were then blocked with 2% normal donkey serum for 2 h at room temperature (RT) and reacted with antiBETL1 or antiBETL2 antisera, or the corresponding pre-sera at 1:500 dilution. Reacted primary antibodies were detected with biotin-conjugated anti-rabbit goat antibody diluted 1:750 (Sigma) and then with Extravidin-peroxidase (Sigma) solution at 1:800 dilution. Finally, positive reaction was developed using SIGMAFAST™ DAB with Metal Enhancer (Sigma) until a gray-black precipitate was clearly visible on the sera-reacted slides. The sections were stained post-detection with 0.025 % Azure B in phosphate buffer pH4 for 3 min, washed in abundant distilled water, mounted in DEPEX and photographed as described in Muñiz et al. (2006).

## Results

### Collection of RNA from wild type and mutant sibling developing kernels

**Figure 1:**
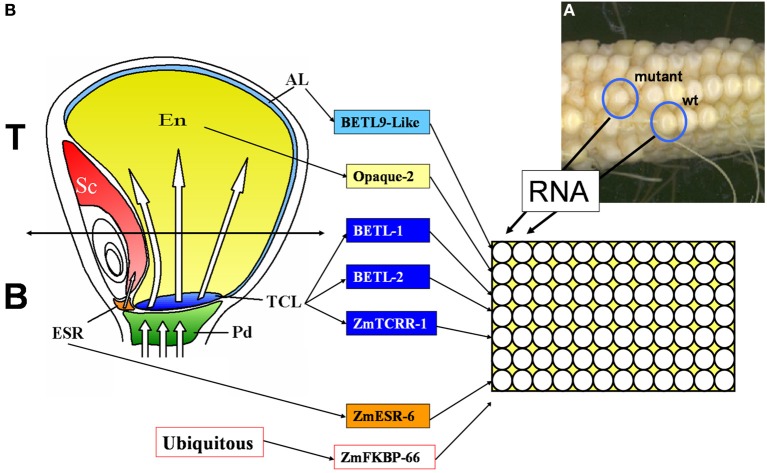
**Diagram describing the setup of the qRT-PCR screening used in this work. (A)** Image of a cob segregating for WT and miniature mutant phenotypes, source for the current study. **(B)** Sagittal section of a 15 DAP maize kernel showing the different tissular domains it contains. Pd, pedicel; ESR, embryo surrounding region; TCL, transfer cell layer; Em, embryo; En, endosperm; Al, aleurone. The markers used in this study and the tissues they mark are indicated in the central panel. The right panel shows the organization of the PCR plates, total RNA was extracted from sibling mutant and wild type (WT) kernels and analyzed in parallel columns, each row was used for a tissue specific marker or the ubiquitous control used for data normalization.

A visual screening among 25,000 maize lines of a *Mu* transposon-mutagenized collection allowed identification of 600 lines showing alterations in the development of the endosperm. These lines were classified as *min* (miniature) or *udve* (undeveloped with embryo) (Figure 1A). F2 seeds from 101 mutant lines were planted in the field to produce the material used in the present work. Immature cobs were opened for visual inspection starting at 13 days after pollination (13 DAP). In most cases WT and mutant kernels could be un-ambiguously distinguished in segregating ears 13–15 DAP. Kernels were collected at the youngest possible distinctive developmental stage, frozen in liquid nitrogen and stored at −80°C.

On average, 200 μg total RNA was obtained from the WT kernels and 80 μg from the mutant kernels, although wide variation was observed for the mutant samples among the different lines. The quality of the RNA was analyzed by formaldehyde-gel electrophoresis of aliquots of 3 μg (not shown); only samples showing intact ribosomal RNA species were used for real time PCR.

### Design of real time PCR assays for developing endosperm expression domain specific markers

Marker genes were selected for different expression domains within the developing endosperm (see Figure 1B for a graphical representation of the domains analyzed). The expression pattern of all the markers used in this study has been characterized in detail, including *in situ* hybridization studies. The list includes 4 markers for the transfer cell layer: *BETL-1* and *BETL-2* (Hueros et al., 1999), *ZmTCRR-1* (Muñiz et al., 2006), and *ZmMRP-1* (Gómez et al., 2002, 2009); 1 marker for the embryo surrounding region: *ZmESR-6* (Balandín et al., 2005); 1 marker for the aleurone: *BETL-9like* (our group, see Royo et al., under review) and 1 marker for the starchy endosperm: *Opaque-2* (the expression of the gene was analyzed by *in situ hybridization* in Dolfini et al., 1992). As an internal control for data normalization, we selected the ubiquitously expressed gene *ZmFKBP-66* (Hueros et al., 1999). Different primer sets were tested for each gene (data not shown) until a primer pair was identified (Table 1) satisfying the following criteria: 1. No PCR product was amplified from 50 ng genomic DNA, using the same PCR conditions designed for the real time RT-PCR assays, 2. In the absence of PCR product, no primer dimers were formed, and 3. The efficiency of the amplification, as determined by the standard curve method, was similar for all genes, and approached 1 (not shown).

**Table 1:** **Summary of primers used in the transcriptomic analysis**.

**Gene**	**Amplicon size (bp)**	**Primers (5′-3′)**	**Expression domain**
*BETL-1*	93	CAGCACAATCGTCGCGCTT	Transfer cells
		TTCTTGGGTTTCCCGATGC*AGC	
*BETL-2*	115	TGCACGCACAACAAGTG*GGC	Transfer cells
		AGCATGGCCCGTCGTCATT	
*ZmMRP-1*	120	GACTACAGATGAGCACAG*GAATTTC	Transfer cells
		GCATGGCTAGAGATCTGCA	
*ZmTCRR-1*	106	ATTGGAATTCTTAGATGCG*AAC	Transfer cells
		CGATTC*CTTCACTTCCCTAA	
*ZmESR-6*	90	GCCATAACCATGCCGTCCT	ESR
		TGCAGACGCATCCATTC*CGA	
*BETL-9like*	283	CTATGTTTGCCATAGGCTCTCATGC	Aleurone
		GCTGGAACCTTGTAGC*TTCCG	
*Opaque-2*	158	AGAACTGGAGGACCAG*GTAGC	Starchy
		CCTCTCCCATCTTCAC*CTTA	endosperm
*ZmFKBP-66*	117	GGGTGCTGTTGTTGAAG*TCA	Ubiquitous
		GCAATAA*CTTCCTCTTCATCG	

The table presents gene name, expected amplicon size, primer sequence and represented tissue domain. The asterisks indicate the intron/exon boundary.

The ability of the selected primer pairs to detect domain specific expression was tested in real time RT-PCR experiments using as template total RNA extracted from the upper (Top in Figure 1B) or lower (Bottom in Figure 1B) halves of WT kernels collected at 10 DAP. As shown in Figure 1B, the upper part of the kernels does not contain transfer cells and very little embryo surrounding region, whilst both the upper and lower halves should contain roughly equivalent amounts of aleurone and starchy endosperm tissues.

**Figure 2:**
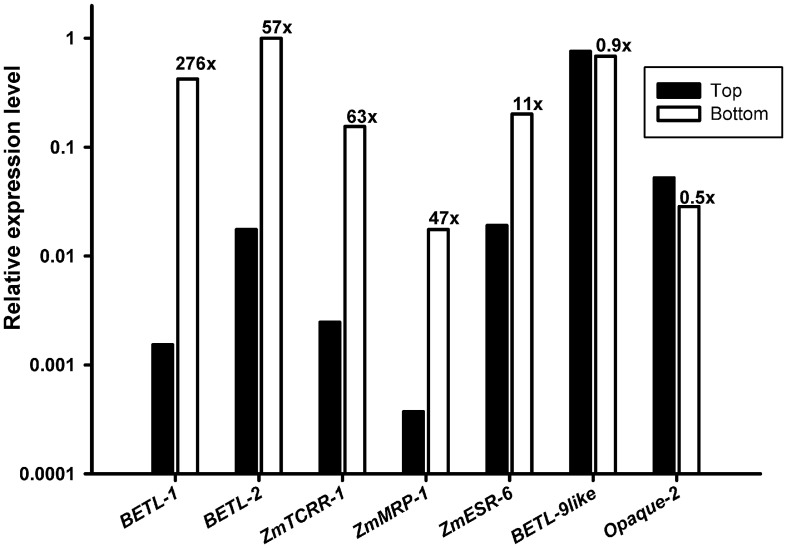
**Expression analyses of the marker genes in wild type kernel halves.** RNA from the upper (**Top**, black bars) and lower (**Bottom**, empty bars) halves of 10 DAP wild type kernels was used as template for the markers indicated in the X axis. The Y axis scale is logarithmic (base 10). The ratio **Bottom/Top** found for each marker is indicated above each empty column.

Consistent with the distribution of tissues within the seed, the results (Figure 2) showed a higher accumulation of transcript in the bottom samples, with a difference of over 1 order of magnitude, for the transfer cell and ESR specific markers. Equivalent amounts of transcript were detected, however, in Top and Bottom for the aleurone and starchy endosperm specific markers.

### Real time PCR analyses

**Figure 3:**
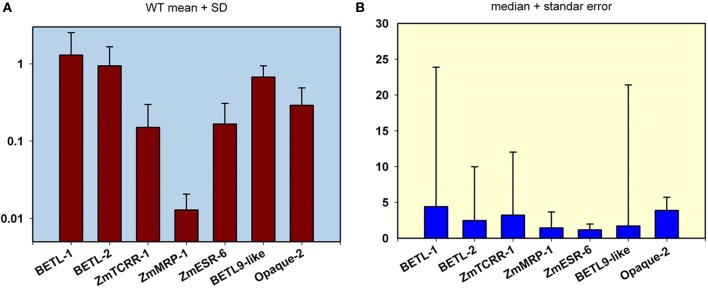
**Transcript level quantification in the wild type (WT) and mutant kernels from the 101 mutant lines analyzed in this study. (A)** Expression analyses in WT kernels. The mean value and standard deviation from the analysis of the 101 WT samples are indicated for the 7 markers used in this study. The scale in the Y axis is logarithmic. Values are referred to the highest expressed gene (BETL-1). **(B)** Representation of pooled WT/mutant relative expression levels. The median value and standard error from the analysis of the 101 WT/ mutant pair samples are shown for the 7 markers used in this study. The Y-axis scale is linear.

For the real time PCR analyses of the selected lines, the scheme outlined in Figure 1 was followed. We designed an analysis setup for pairs of total RNA samples from immature WT and mutant sibling kernels in parallel columns of 12 × 8 well PCR plates. PCR primers specific for the seven tissue-specific markers and the ubiquitous control were distributed in each of the 8 rows of the plate (see the schematics in Figure 1B). The analyses of the expression data for the WT kernels from the 101 lines (Figure 3A), shows that the markers expression level ranges from highly expressed (*BETL-1*, *BETL-2*, and *BETL-9like*) to very lowly expressed (*ZmMRP-1*), encompassing nearly two orders of magnitude. The expression levels of *ZmTCRR-1*, *ZmESR-6*, and *Opaque-2* were intermediate between the other two groups. The variation in the expression level of the markers among the WT samples from different lines was moderate, and very likely reflects differences in the maturation stage of the different ears at the time the kernels were collected. The low expression level found for *ZmMRP-1* could compromise the sensitivity of the WT/mutant comparisons and we therefore decided not to include this marker among the primary criteria for selection of putative transfer cell mutant lines.

A pooled comparison of the effect of the different mutations on the expression of the markers reflects a wide range of variation among the lines and among markers (Figure 3B). The analysis of the structure of this variation indicates that most lines show only a moderate decrease in the expression of the domain markers. As a result, the median values for the different markers' WT/mutant ratio are below 5, being as low as 1.16 in the case of *ZmESR-6*. This gene and *ZmMRP-1* display the lowest median WT/mutant variation, possibly confirming the aforementioned discrimination limit for lowly expressed genes. In the case of *ZmESR-6*, which has a higher expression than *ZmMRP-1*, the limited extension of its expression domain at this stage may contribute to the low WT/mutant ratio found. On the other hand, in a small number of lines the expression ratio WT/mutant for certain markers indicated a variation of several orders of magnitude (note the long error bars in Figure 3B for *BETL-1*, *-2*, *ZmTCRR-1*, and *BETL-9*). Most probably, in these lines the mutation has altered the early differentiation of a certain cell type leading to a significant reduction of the expression levels of the corresponding markers.

**Figure 4:**
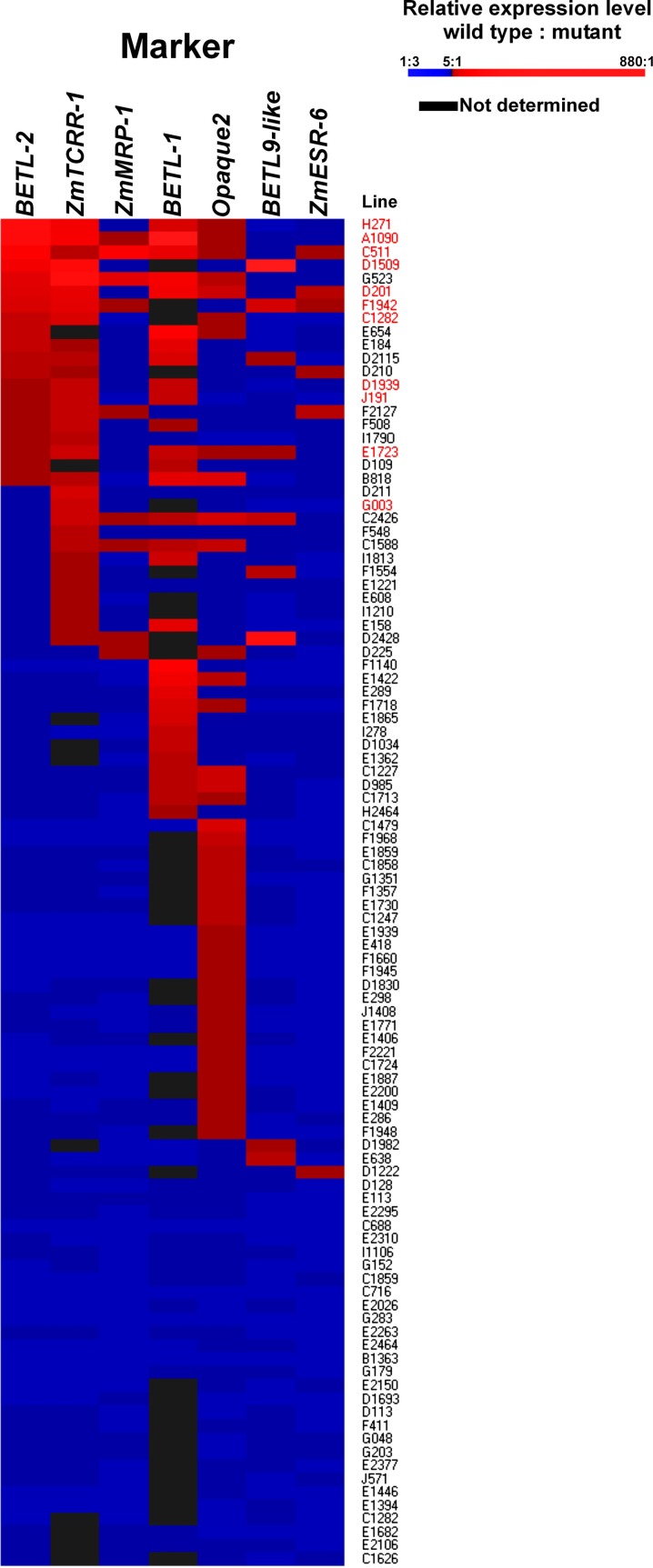
**Organization of the 101 lines according to marker expression ratio.** Lines are ordered according to the WT/mutant expression ratio, first for *BETL-2*, then for *ZmTCRR-1*. WT/mutant ratios were then converted into color intensities to facilitate graphical representation, according to the scale shown.

The expression ratios were converted into color variations to visualize the expression patterns of the different markers and lines (Figure 4), the lines were then ordered according to the WT/mutant ratio displayed for *BETL-2* and *ZmTCRR-1* (the numeric values the figure is based on are supplied as Supplementary Table S1). These markers were chosen as first and second hierarchical parameters for the evaluation of the lines, regarding the putative absence of the TC domain, as both of them were reliably measurable by qRT-PCR. In this way, 11 lines (labeled in red in Figure 4), with differing severity in the alteration of the TC marker expression levels, were selected for further characterization. Surprisingly, in the case of *BETL-1* no expression could be detected in 36% of the lines in either WT or mutant kernels. This result discarded the expression data recorded for this marker as the primary parameter for the selection of candidate lines and suggest that the expression of this marker might be influenced by environmental factors.

For 29 lines, no significant difference in the expression level of the markers in the WT and mutant samples was found. The remaining 72 lines showed a WT/mutant ratio >5 for at least one of the markers (Figure 4).

When the aleurone and transfer cell specific markers were examined, only 10 (for *BETL-9like*) or 30 (for *BETL-2*) lines showed a ratio WT/mutant higher than 5, although this ratio reached levels above two orders of magnitude in particular cases. This contrasts with the situation observed for *Opaque2*, which showed a ratio >5 in 42 (58%) of the lines showing any variation, and was the only marker affected in 25 cases. The decrease in the expression of the *Opaque2* marker in the mutant kernels was, however, moderate; for the majority (31 lines) the ratio WT/mutant ranged between 5 and 10 and was never higher than 30. The ESR marker showed the least affection, with WT/mutant ratios higher than 5 only in 6 cases and a quite moderate range from 5 to 11 fold.

The 11 lines selected were then rechecked by qRT-PCR on RNA extracted from sibling seeds. The qRT-PCR analyses showed the results of line C1282 were not reproducible on the new RNA samples (no significant differences were found in this round of tests, not shown), and the line was removed from subsequent studies.

### Histology and immunoanalyses of selected lines

**Figure 5:**
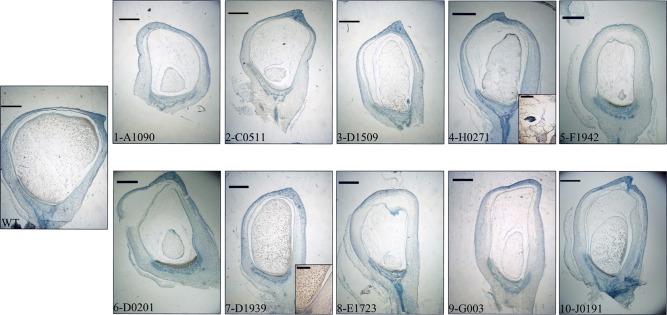
**Seeds from each line were sagittally cut and sectioned at 8 μm thickness.** Low magnification images (15×) are shown for comparison of general kernel structure and size. The WT kernel shown corresponds to line A1090. Scale bars represent 1 mm in main panels and 50 μm in insets.

**Figure 6:**
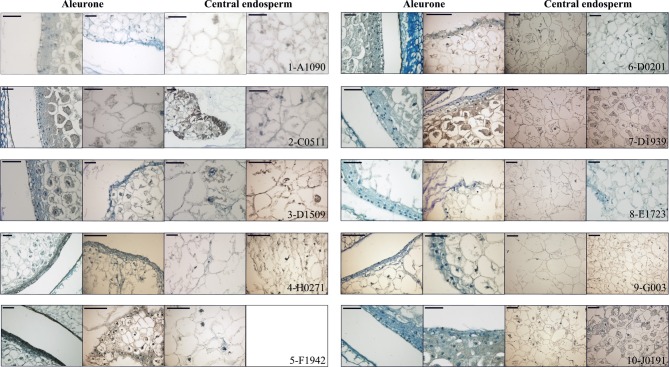
**Aleurone and central endosperm structure.** Sections of WT (first and third column) and mutant (second and fourth column) 15 DAP kernels from each line are presented, showing magnifications of the aleurone and starchy endosperm cells. Sections are stained with azure B. Scale bars represent 50 μm. Note that for line F1942 only one mutant image is shown, as the reduced endosperm size allows detailed visualization of central endosperm and aleurone in one frame.

**Figure 7:**
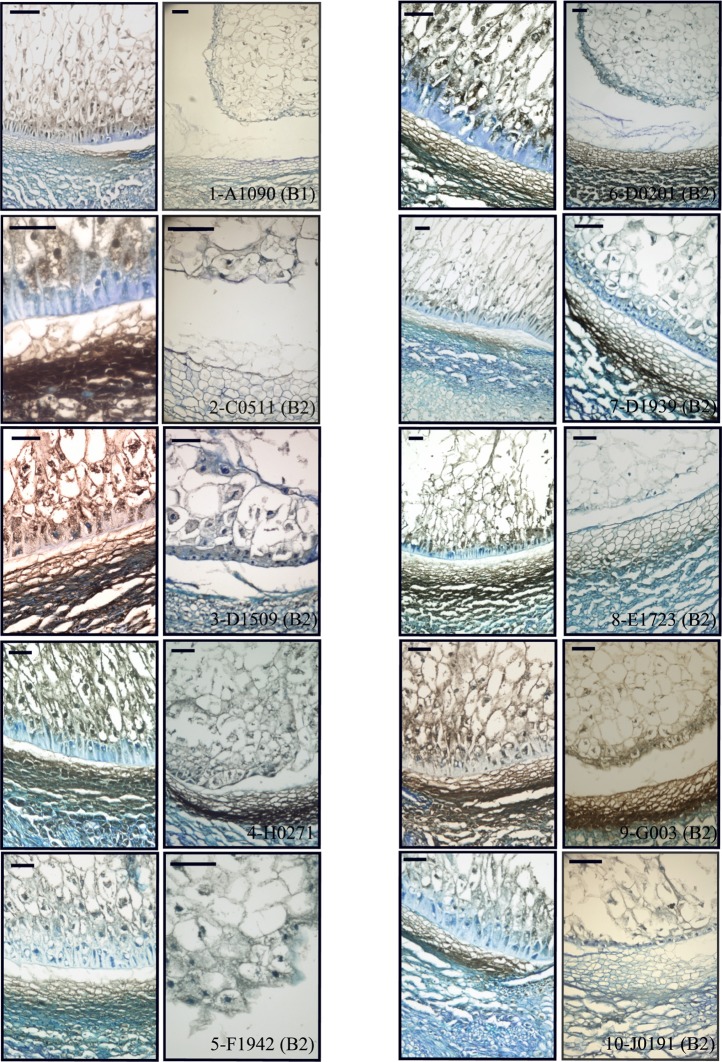
**Basal endosperm structure and immunodetection of BETL peptides.** Sections of WT (first and third column) and mutant (second and fourth column) were reacted with antiBETL1 or antiBETL2 and counterstained with azure B. Scale bars represent 50 μm.

As a way to obtain an anatomical perspective of the effect of the mutations under study, investigate what their effect on seed structure is, and validate the qRT-PCR analysis' ability to predict specific tissue alterations we prepared optical microscopy sections of 15 DAP WT and mutant seeds for histological observation. Additionally, we performed immunolocalization of several TC specific proteins to check correspondence between transcript and protein levels and examine protein localization in this compartment. We used polyclonal sera generated against recombinant BETL-1 or BETL-2 peptides. These proteins are secreted from the transfer cells to the placento-chalaza, forming a continuous layer in the seed-mother plant interface (Hueros et al., 1995; Serna et al., 2001), so potentially they offer an indication of the tissue's ability to perform some of its functions. As expected, both antibodies labeled the transfer cell area and the pedicel directly opposite to the TC in sections of 15 DAP WT seeds (Figure 7, top left panel. Only BETL-1 on A1090 is shown). We provide now a summary of the results for each mutant line focusing on three aspects: macroscopic alterations and size, transfer cell domain markers (both gene expression and protein accumulation) and histological features:

**A1090:** The 15 DAP endosperm is highly reduced in size relative to the WT, which at this stage replaces the nucella almost completely (Figure 5, panel 1). The surface of the endosperm, where the aleurone is located, is formed by differentially stained cells which are much altered in appearance; these cortical cells are flattened and interspersed with starchy endosperm cells. Accumulation of starch in the central endosperm is not significantly affected, together with the general appearance of the storage cells, although their size is somewhat smaller (Figure 6, panel 1). The cells at the base of the endosperm, where the transfer layer should be, are also flat and miss their characteristic cell wall ingrowths (Figure 7, panel 1). In summary, in this line all the superficial cells seem to have adopted a similar fate, different from storage cells but without distinctive features of aleurone and transfer cells, although resembling an immature aleurone layer. The qRT-PCR analysis of this line shows severe decreases in markers for the TCs and mild reductions in those for aleurone and starchy endosperm. The immunodetection of TC marker peptides in 15 DAP kernels reveals, in agreement with the qRT-PCR results, a very low signal from BETL-1 and -2 from the basal endosperm in mutant seeds, while they are readily detectable in WT siblings (Figure 7, panel 1: only BETL-1 detection is shown). In this line TC molecular markers faithfully reflected the histological alterations, while the degree of modification of other altered markers (*Opaque-2*, *ZmESR-6*, *ZmMRP-1*, or *BETL-9like*) did not correlate so well with the anatomy.
**C0511**: In this line kernel size is much reduced and nucellar tissue still occupies most of the caryopsis (Figure 5, panel 2). Starch deposition is much delayed in the peripheral endosperm when compared to WT, although this difference is not so significant in central endosperm at this stage. Tissue organization within the endosperm is lax, the storage cells being the only clearly recognizable structure. There are, however, patches of aleurone-like cells, with a cubical or prismatic morphology, which surround the starchy endosperm (Figure 6, panel 2). The transfer cells are absent or not properly developed for morphological recognition (Figure 7, panel 2). The transcript levels for transfer cell and ESR genes in this line are reduced between 1 and 2 orders of magnitude in mutant kernels, and aleurone and endosperm markers are also reduced, indicating a general underdevelopment of the seed. The strong sub-expression of *ZmMRP-1* might explain the down-regulation of the TC markers under its transcriptional control, and strongly corresponds with the severe alterations in the transfer cell identity. The immunological detection of BETL-2 shows an irregularly distributed signal at the basal cells and none at the pedicel of the mutant, while the immunostaining is strong in WT material (Figure 7, panel 2). In this line the molecular characterization reflects the anatomical events quite faithfully, as the basal endosperm markers' severe alteration and the endosperm *Opaque-2* reduction are validated by the absence or erroneous development of transfer cells and an apparent delay or arrest in storage tissue function.
**D1509**: The morphological study of mutant seeds shows that their size is less reduced than in other mutants in this work, with the endosperm occupying about half of the nucellar space (Figure 5, panel 3). Apical and basal poles are clearly defined, although the internal endosperm organization is quite lax. Sub-aleuronal starch accumulation, visible in WT kernels is absent in the mutant, although the internal endosperm seems well-differentiated. It can be clearly distinguished from peripheral tissues, which in the case of the aleurone shows a flattened, collapsed appearance and stains more intensely than the inner cells (Figure 6, panel 3). Likewise, the transfer cells are not organized in a “palisade” layer, but rather as a flat, discontinuous row of dense cytoplasm-containing cells, compressed by the internal endosperm, which occasionally invades the layer. The intermediate, elongated transmitting cells seem to be absent in the mutant (Figure 7, panel 3). qRT-PCR analysis of this line shows acute differences between WT and mutant kernels regarding *ZmTCRR-1*, *BETL-2*, and *BETL-9like*, which suggest abnormalities in the outermost endosperm layers (*BETL-1* was not analyzed in this material as apparently the line lacks this gene). The TC master regulator, *ZmMRP-1*, is however, less affected than its targets, suggesting an independent cause for their down -regulation. *Opaque-2* and *ZmESR-6* are reduced to a minor extent (about 4 and 5 times, respectively). The immunolocalization of BETL markers (BETL-2, as the line doesn't show any detectable BETL-1 expression) shows a strong signal in the transfer layer and the underlying pedicel of WT individuals, while it is much reduced in the mutant, matching the RNA expression data (Figure 7, panel 3). The molecular definition of the mutant is consistent with its tissular appearance, anticipating the superficial disorders that can be found under optical microscopy.
**H0271**: Morphologically, the mutant endosperms are small in size (about 1/3 of the WT at this stage) and elongated in shape, reminding of a younger developmental point (Figure 5, panel 4). The embryo encapsulation within the endosperm is still clearly observed (inset in panel 4) and the tissue seems disorganized and mixed with both the starchy endosperm and extended aleurone around the embryo. The starchy endosperm is quite similar in both genotypes, in the central area as well as in the periphery, with very little starch present. Storage cells are however smaller in the mutant. The aleurone layer is flattened and the cells show a dense cytoplasm, except those on the outer germinal surface which show a more rounded, and regular shape (Figure 6, panel 4). The transfer cell layer is apparently missing or totally altered in its morphology, as the cells in this area are irregular in shape and devoid of cell wall ingrowths (Figure 7, panel 4). The expression analysis of this line indicates a severe reduction in TC transcripts (from 24 times for *BETL-1* to almost 150 for *BETL-2*) in the mutant kernels, while other regional markers are only slightly affected (this is the case of *ZmESR-6*, which is reduced by approximately 50% in the mutant) or even elevated. The immunological study shows no detection of BETL-1 peptide (not shown). However, in contrast to the extremely low RNA levels, accumulation of BETL-2 can be detected at the basal endosperm and pedicel (Figure 7, panel 4). The apparent disagreement in BETL-2 transcript and protein accumulation levels notwithstanding, the transfer cell developmental alteration visible under the microscope was in fact detected by the molecular tests.
**F1942**: The mutant endosperm is reduced to a minute fraction of the normal size (in fact the smallest one of the mutant lines), being formed by only a few tens of poorly differentiated cells that seem randomly organized (Figure 5, panel 5). The mutant central “starchy endosperm” cells are bigger and more regular in size than the peripheral ones (Figure 6, panel 5). The endosperm surface is not cytologically regular, and internal cells occasionally occupy it. There are no obvious aleurone or transfer cell layer and no starch deposition can be found. All tissue markers (except *BETL-1*, which is not detectable in WT kernels either) are down to a certain extent in the mutant kernels of this line, from 4.5-fold for *Opaque-2* to over 30-fold for *BETL-2*, indicating a general alteration in development. Anti-BETL-2 labels the whole periphery of the endosperm, concentrating on cells of small size and flat or elongated structure (Figure 7, panel 5), although the signal is weak and almost indistinguishable from general background. The line's extensive abnormalities reflect well on the molecular results, although the expression analysis does not directly indicate the severity of the alterations, when compared to other lines.
**D0201**: The size of the mutant endosperm at 15 DAP is about one-third of the WT sibling, with a pear-shaped morphology (Figure 5, panel 6). The aleurone is deformed with occasional invasion from the internal cells, and there is no apparent starch accumulation in the subaleurone or in the small cells that constitute the central endosperm (Figure 6, panel 6). The transfer layer showed a strikingly bi-phenotypic morphology in this mutant. The cell layer is composed by thick-walled WT-looking transfer cells at the germinal half, and storage type-looking cells at the abgerminal end. Cells in this area are irregular in shape, not corresponding in morphology to neither aleurone nor transfer cells (Figure 7, panel 6). The expression analysis of the mutant kernels shows reduced expression of all marker genes, except for *ZmMRP-1* and *BETL-9like*, the aleurone transcript. The detection of BETL-2 in this line showed signal in the pedicel cup, as expected, but only in the germinal half of the transfer layer and less intense than in the WT (Figure 7, panel 6. A lower magnification is shown to include the pedicel in the mutant). In summary, the altered morphology of the mutant seed is reflected on the expression analysis.
**D1939**: This line displays a remarkable difference with the other lines analyzed. The endosperm size is fairly normal in this line, about 2/3 of the WT, with a clear morphological differentiation between starchy endosperm and superficial cells (Figure 5, panel 7). However, the starch deposition pattern appears altered; the central endosperm accumulates more starch than the WT, and this extends to the subaleurone, especially along the germinal and abgerminal sides of the endosperm instead of the characteristical upper dominance typical of WT kernels at this stage (Figure 6, panel 7; inset in Figure 5, panel 7). The surface of the endosperm is formed by a layer of nearly flattened cells which become slightly more prismatic in the transfer area, acquiring also engrossed cell walls. These cells lack, however, the characteristic wall ingrowths expected in transfer elements, and they resemble an engrossed aleurone or an intermediate state between aleurone and transfer cell fate. The conductive tissue immediately over the transfer area lacks its typical centripetally elongated shape and invaginations, and is morphologically indistinguishable from the upper endosperm area (Figure 7, panel 7). Expression abnormalities in the mutant focus on the transfer cell markers, and in a relatively moderate manner (from 14-fold in *BETL-1* to 1.6-fold for *ZmMRP-1)* while other areas show less effect (just 3.3 times for *Opaque-2*), and the marker for aleurone cell fate, *BETL-9like*, is in fact unregulated over 3 times. The expression analysis would in summary indicate a state of mild alteration or developmental delay for the mutants, affecting specially the base of the endosperm in this line (as compared to others in the collection). The immunolocation of BETL-1 and -2 shows agreeing results, with no detectable signal from BETL-1 and a faint one, mostly in the pedicel from BETL-2 (BETL-2 is shown in Figure 7, panel 7). As suggested by the molecular study, most the alterations focus at the transfer cells, while the aleurone and starchy endosperm look relatively unaffected.
**E1723**: The anatomical appearance of the mutant seeds is deeply affected in this line. The endosperm is reduced to a small mass of cells which doesn't cover the whole pedicel surface and shows no defined polarity (Figure 5, panel 8). There is no apparent tissue organization, as the whole mass is formed by storage cells diminishing in diameter close to the surface of the endosperm, where many cells are collapsed (Figure 6, panel 8). The mutant seeds displayed significant downregulation of the markers under study, with the exception of *ZmMRP-1* and *ZmESR-6*, although the WT/mutant ratios are lower than for other lines in this work. The values range from 18 (*ZmTCRR-1*) to 3.6 times (*ZmESR-6*). The immunological markers tested in this mutant are either undetectable (BETL-1) or faint and limited to the areas where the protein accumulates at higher levels in WT seeds (BETL-2 at the pedicel, Figure 7, panel 8). The developmental alterations in this line were correctly predicted by the molecular analysis, although their severity is much greater than what can be found in other lines with greater WT/mutant expression ratios.
**G003**: As in other mutants in this study, the endosperm is reduced in size (about 1/3 of the WT), although its general axial organization is normal (Figure 5, panel 9). Tissular domains are clearly differentiated and occupy their regular positions, with the periphery being clearly distinguishable from the central endosperm. The cells in this tissue are smaller in the mutant, and show lower or no starch deposition. The aleurone is formed by a layer of 1–3 thick cells that become wider over the upper endosperm portion. The walls of these cells are more intensely stained than those in the WT, possibly indicating a greater thickness (Figure 6, panel 9). The transfer cells, conversely, seem to have a thinner, less stained than WT appearance, and their shape varies from stretched to almost round (see Figure 7, panel 9). The area on top of them, the transmitting tissue, is indistinguishable from the starchy endosperm, while the WT displays the characteristical axial elongation in this area. The analysis of domain markers in this line shows that the mutant individuals do not differ much from the WT as far as the ESR (*ZmESR-6* ratio approx. 0.7), the aleurone (*BETL-9like* ratio approx. 1) and the starchy endosperm (*Opaque-2* ratio approx. 2.9) are concerned. The transfer cell markers are affected by the mutation to a different extent, as *BETL-2* is slightly down-regulated (about 3 times) and *ZmTCRR-1* is down by over one order of magnitude (17.5). *BETL-1* expression is hardly detectable in this line. *ZmMRP-1*, the transcription factor that regulates the expression of *BETL-1*, -2 and ZmTCRR-1 is also slightly down-regulated. The immunolocalization experiments showed that BETL-2 accumulation concentrates mainly at the pedicel, with a patchy location at the endosperm base (Figure 7, panel 9). This mutant line presents a comparatively mild phenotype, as could be expected from the molecular analysis, in which only *ZmTCRR-1* indicated alterations in the transfer cell area.
**J0191:** The endosperm size is reduced approximately by half in the mutant, and the starch deposition pattern within the endosperm differs significantly from the WT, as abundant starch granules accumulate at the germinal side and extend into the central endosperm (Figure 5, panel 10). In fact starch storage seems more advanced in the mutant, as the cells are more densely filled with granules. The cells in peripheral positions display a thick cell wall all around the endosperm, although they differ in shape; the basal cells are rectangular, almost cubical in front of the phloem terminals, and display a dense cytoplasm. The cells in the aleurone layer are flattened along the whole layer. The adgerminal subaleurone displays a dense cytoplasm, filled with starch (Figure 6, panel 10). The main effect at the transcriptional level of this line's mutation shows on the transfer cell markers, which are down regulated between 7 and 15 times (Figure 4), pointing at an altered development of the transfer cell area. Other transcriptional domains are relatively unaffected, as values for *ZmESR-6* and *Opaque-2* are even higher in mutant seeds than in WT, while the aleurone marker is down-regulated only twice. BETL-2 is detected at both the basal endosperm layer and the pedicel, although significantly reduced in quantity. The signal at the endosperm is limited to the tissue immediately above the altered TCs, while the WT kernel shows abundant signals at the pedicel and the basal endosperm (Figure 7, panel 10). The overall mutant appearance in this line is fairly regular, with the exception of an apparent increase in starch accumulation. This observation, together with the increased *Opaque-2* expression suggest an advanced development for the storage tissue, while the transfer cells appear underdeveloped and not fully differentiated from the aleurone.

## Discussion

Large mutant collections in a variety of model organisms are a powerful tool to understand the impact of the genetic component in the functional biology of the individual. However, as phenotype scoring is the access gate to the study of the underlying genetic mechanism (Hotz et al., 2006), a comprehensive and thorough screening process becomes the key to their use. Analyzing or even finding phenotypes related to the process or tissue of interest is especially difficult when these are not easily accessible to assay (Kielczewska and Vidal, 2006). A wide variety of approaches has been used, from classical bare-eye observation (Kuromori et al., 2006) to complex functional *in vivo* and *in vitro* assays (Burrack and Higgins, 2007). The development of molecular tools at high throughput scale has allowed to include metabolome (Benning, 2004), proteome and transcriptome analysis as a part of a mutant's phenotypic description (Domoney et al., 2006). The availability and variety of markers greatly facilitates the isolation and study of new mutant lines, although their development and design may be cumbersome (Guitton et al., 2004). It is not unrealistic to expect that markers based on genes with developmentally regulated expression in a given tissue or cell type are somehow involved in determining its identity (Li and Wurtzel, 1998; Kubo et al., 2005); thus, the growing information about gene expression timing and localization collected in the bibliography and public databases allows to design directed transcriptomic screens to study particular processes or tissues, even when these are not easily accessible. This work has a dual purpose: we have designed and tested a molecular kit for the screening of a maize seed mutant collection and the dissection of mutant phenotypes affecting seed development, based on a set of genes previously identified as specific of particular kernel compartments. Secondly, we report the characterization at the molecular and histological levels of a series of maize mutants displaying seed phenotypes.

The transcripts used to “label” the transfer cell area, embryo surrounding region and aleurone have been isolated and described by our group, and detailed data exist about their expression site, developmental regulation and in some instances functional hierarchy. *ZmESR-6* encodes a defensin-like peptide that has shown antimicrobial activity in *in vitro* test (Balandín et al., 2005). *BETL-1* and *-2* have currently no known function, although *BETL-2* may also be involved in seed defense, as *in vitro* tests have shown bactericidal properties for the peptide (Serna et al., 2001). *ZmTCRR-1* shows sequence similarity to plant response regulators and is proposed to participate in signal transduction in the seed (Muñiz et al., 2006). The three TC transcripts have been shown to be transcriptionally regulated by *ZmMRP-1*, a R1 MYB-type transcription factor which is expressed in the developing kernel since early stages and whose activity suffices to confer transfer cell features to aleurone cells (Gómez et al., 2002, 2009). *ZmBETL-9like* encodes a peptide of unknown function which is expressed along the aleurone layer, complementing the surfacial expression pattern of its paralog ZmBETL-9 (see Royo et al., under review). Together with *Opaque-2*, isolated over 16 years ago, they may constitute a valuable set of tools to describe the anatomical and developmental state of the maize kernel and predict the alterations to be found within the seed structure. We validated their use as indicators of presence/state of development of a specific tissue in qRT-PCR by comparing the relative values obtained in the analysis of upper (formed by aleurone and starchy endosperm) and lower (aleurone, starchy endosperm, transfer cells and ESR) halves of WT seeds. Previous studies through Northern blot and *in situ* hybridization have shown that these BETL and ESR transcripts are undetectable by these techniques in the upper endosperm, while aleurone and starchy endosperm markers distribute approximately evenly (Hueros et al., 1999; Gómez et al., 2002; Balandín et al., 2005; Muñiz et al., 2006; Dolfini et al., 2007). The differences in the values obtained are in all cases in agreement with previous data, showing over 1 order of magnitude difference in their accumulation in bottom over the top half for the basal specific genes, while those expressed in the aleurone and starchy endosperm are roughly comparable in both samples. The value of the bottom/top ratio is highly dependent on the abundance of the different transcripts, as the quantification threshold of bottom-specific genes for the top samples is always close to the detection limit of the technique. This highlights an otherwise expected technical consideration: genes with low expression levels display a lower sensitivity for mutant discrimination in this strategy, as the screening is based on the comparison of expression strength in WT and prospective mutants.

Once the design of the qRT-PCR experiment was set, it allowed for the rapid transcriptomic screening of over 100 lines for markers representing 4 compartments within the endosperm. The fact that no expression for *BETL-1* was detectable in up to 1/3 of the lines can be explained by the genetic structure of the mutant collection, which comes from a number of crossings of different Mu-bearing maize parents. A variation in the number of *BETL-1* loci among maize lines, probably due to active transposable elements at their location, was already described in Hueros et al. (1999).

The application of the directed transcriptomic screening to WT individuals from the mutant collection showed very minor variations in the expression of the different markers, supporting their use as reliable indicators of WT structure. In 29 lines (28.7% of the analyzed genotypes) none of the markers presented alterations. Pending a morphological study of these lines, (which is beyond the scope of the present paper) this would point to an origin of the macroscopic phenotype (*min* or *udve*) not associated to significant identity alterations in the endosperm domains studied. Alternatively, a different set of marker genes might be able to detect alterations in these tissues that have gone undetected in our panel. If this is the case, fine-tuning the identity of the markers or increasing their number and their relevance for the tissue or developmental event under study may yield a higher correlation between the molecular and microscopical approaches. In our case, we screened the starchy endosperm using a marker essential for its storage function, and the transfer layer with four markers. The hierarchical relationship among *ZmMRP-1* and the other TC transcripts might however limit the range of exploration, and more diverse, independent markers might be desirable.

The analysis of WT vs. mutant seeds showed that the transcriptional alterations were under 1 order of magnitude for most of the lines, although significant outliers could be found. These severely altered lines are potentially of great interest to study the effect of certain cell type alteration or absence on the seed development. The highest variation (not including *BETL-1* due to the afore mentioned reason) was found in *BETL-9like* expression, which was reduced only slightly in most of the lines but displayed strong reductions of up to 885 times in others (Figure 4).

The transcriptome analysis allowed clustering of the 101 maize lines in quantitative categories based on the degree of alteration of the different markers (Figure 4). We have found, as expected, significant correlations in the reductions of the different TC markers, indicating that the mutations found in the collection disrupt the tissue's developmental program in early stages. However, as most of these genes reach their peak expression around 11 DAP, it would be highly informative to have an earlier marker to pinpoint more precisely the developmental effect of the mutation. This adds in fact a new analytical power to the quantitative molecular dissection in cases where a number of genes affecting a given process or tissue are known. The expression analysis of a set of selected genes might show in which point of development the case under study is affected, by determining the earliest gene with altered expression. Our analysis also shows that for these particular mutant phenotypes (miniature and undeveloped with embryo), *Opaque-2* is the most commonly affected marker, which is coherent as the most conspicuous feature in both cases is a reduced seed size, and this is mainly the result of starchy endosperm growth. The analysis of the mutant collection points to a developmental organization along the top/bottom axis and surface/center gradient (Gruis et al., 2006), as most of the lines significantly affected in the ESR also display a TC molecular phenotype (5 out of 6), and transfer cell defects are present in 7 of the 9 aleurone-affected mutants. The marker for central endosperm fate, on the other hand, behaves quite independently from other seed areas, as only 16 of the 41 *Opaque-2*-altered lines show TC, ESR, or aleurone defects.

The transcriptional analysis has allowed us to select lines displaying transcriptional defects in the transfer cell markers to a varying extent (H0271, D1939, J191, G003), surface tissues (D1509) or TC plus starchy endosperm (A1090, C0511, D0201, F1942, E1723), and rechecked the marker expression using qRT-PCR on independent seeds (not shown). Both analysis showed good correspondence, except for line C1282. This may be the result of differences in developmental state of the tested material or incorrect phenotyping.

The anatomical analysis of mutant seeds combined to immunolocation allowed us to further check and refine the description of each of the mutants. However, caution must be exercised when interpreting the immunolocation results quantitatively; the technical features of this analysis preclude proper comparison between samples, as WT and mutant seeds have been processed on different slides. The goals of our immunological study are offering an anatomical perspective of the mutants to complement the expression results, and ascertaining whether transfer cell-specific peptides can still be detected in morphologically altered seeds.

All the lines showing only transfer cell marker alterations (H0271, D1939, J191, and G003) display a reduced endosperm size with a generally conserved seed architecture. The transfer cells are misshaped, with a cubical appearance reminiscent of aleurone tissue. This suggests that although the central/peripheral organization is retained, the mutations in these lines affect determination of transfer cell identity, causing an arrest/redirection of the developmental program to another surface cell type. A similar effect was found in the *emp4-1* mutant, in which *VP1* expression can be found in the basal area (Gutierrez-Marcos et al., 2007). The effect of these basal alterations on the overall endosperm size might reflect a deficient uptake of nutrients and a concomitant delay in development (Dolfini et al., 2007).

Mutant kernels of the line D1509 present altered “surface” markers (both transfer cell and aleurone) in ranges from 50 to almost 900 times WT/mutant values. An exception is made of the ESR cells, for which the WT accumulation is just a bit over 4 times the mutant value. The directed transcriptome analysis offers a relatively accurate portrait of the seed's appearance, as in this line both the immunological markers and anatomical analysis indicate a relatively normal internal structure and seed size (albeit slightly reduced) with surface alterations, which include invasion of the periphery by starchy endosperm tissue. This phenotype is similar to *crinkly4* (Becraft et al., 1996) or *cp2-o12* (Becraft and Asuncion-Crabb, 2000), and suggests the mutated gene is involved in surface differentiation. An increasing number of genes have been shown to affect aleurone and general surface specification in maize, uncovering identity mechanisms based on the proper membrane localization and recycling of kinase-type receptors and signal processors (Becraft and Asuncion-Crabb, 2000; Kessler et al., 2002), whose effect is aleurone-specific at the cellular level but affects the whole seed development. The size reduction, common to the previously commented category, indicates the relevance of the surface tissues to properly complete endosperm filling (Dolfini et al., 2007). A point of further interest in these lines is the mutation's effect on other plant epidermal tissues, as common developmental routes have been discovered along the years (Lid et al., 2004). As germination is often compromised in *udve* mutants, embryo rescue approaches may be necessary to determine whether the mutation affects later stages of the plant life cycle (Gutierrez-Marcos et al., 2007).

Five lines in our analysis showed a severe developmental phenotype, with reduced size, complete disorganization of the storage tissue and starch location, and loss of histological identity. This phenomenon of tissue malformation has been previously associated to a misregulation of cell division (Consonni et al., 2005). Such an extent of alteration suggests that genes acting early in the seed development might be affected, as cell identity proceeds in a clonal manner from the coenocyte phase (Becraft, 2001; Olsen, 2001). *Baseless1*, a maternal effect gene acting in the embryo sac causes alterations in the transfer cell layer position and structure, as well as a general disorganization of the endosperm and delay in embryo development since very early stages, which are visually detectable around 10 DAP (Gutiérrez-Marcos et al., 2006). The molecular analysis we used is especially suitable for the detection of this kind of early-effect mutations, provided a set of suitable markers is available. A feature shared by all the lines is a greater tissue differentiation in the germinal area, where both aleurone and transfer cells display more resemblance to their WT counterparts. This is in agreement with a gradual differentiation and maturation of the seed tissues along several axes, as previously proposed by other authors (Becraft and Asuncion-Crabb, 2000).

As a whole, a wide array of processes are candidates to be mutated in this material as the kernel development is affected by external signals, nutrient availability, positional cues and genetic factors, all acting coordinately along time (Berger, 2003; Consonni et al., 2005; Brugiére et al., 2008). Additionally, gene dosage and imprinting affect tissue specification as well (Gehring et al., 2004; Gutiérrez-Marcos et al., 2006).

We believe the PCR-based strategy proposed here is a valuable tool for the early detection and initial characterization of mutants affecting seed development. The availability of genes specifically expressed in different kernel domains and developmental stages allows to describe, from a transcriptomic point of view, the anatomical effect of a mutation even when direct observation of the affected tissues is not feasible (Verza et al., 2005; Laudencia-Chingcuanco et al., 2006). The use of a sensitive technique such as qRT-PCR permits the application of a panel of expression-based markers to determine whether a given cell type/tissular domain is functioning or developing properly, simultaneously quantifying the extent of the alteration, if any. This use of genes discovered by transcriptomic approaches to be tissue or development-specific further extends the array of tools that can be applied in forward genetics programs.

## Conflict of interest statement

The authors declare that the research was conducted in the absence of any commercial or financial relationships that could be construed as a potential conflict of interest.
